# Single cell transcriptome analyses of the developing zebrafish eye— perspectives and applications

**DOI:** 10.3389/fcell.2023.1213382

**Published:** 2023-06-29

**Authors:** Oliver Vöcking, Jakub K. Famulski

**Affiliations:** Department of Biology, University of Kentucky, Lexington, KY, United States

**Keywords:** single cell transcriptome, zebrafish, retina, anterior segment, transgenic

## Abstract

Within a relatively short period of time, single cell transcriptome analyses (SCT) have become increasingly ubiquitous with transcriptomic research, uncovering plentiful details that boost our molecular understanding of various biological processes. Stemming from SCT analyses, the ever-growing number of newly assigned genetic markers increases our understanding of general function and development, while providing opportunities for identifying genes associated with disease. SCT analyses have been carried out using tissue from numerous organisms. However, despite the great potential of zebrafish as a model organism, other models are still preferably used. In this mini review, we focus on eye research as an example of the advantages in using zebrafish, particularly its usefulness for single cell transcriptome analyses of developmental processes. As studies have already shown, the unique opportunities offered by zebrafish, including similarities to the human eye, in combination with the possibility to analyze and extract specific cells at distinct developmental time points makes the model a uniquely powerful one. Particularly the practicality of collecting large numbers of embryos and therefore isolation of sufficient numbers of developing cells is a distinct advantage compared to other model organisms. Lastly, the advent of highly efficient genetic knockouts methods offers opportunities to characterize target gene function in a more cost-efficient way. In conclusion, we argue that the use of zebrafish for SCT approaches has great potential to further deepen our molecular understanding of not only eye development, but also many other organ systems.

## Introduction

The advent of new scientific methods always greatly contributes to discoveries and drives progress. For molecular biologists it could be argued that two of the most recent highly impactful techniques developed have been CRISPR-based gene manipulations and single cell transcriptome (SCT) analyses (Jinek et al., 2012; Xue et al., 2016; Adli, 2018; Adil et al., 2021; Nidhi et al., 2021; Mishra et al., 2022). Both techniques greatly influenced the advancement of science. While the use of the CRISPR-based systems for genetic knockouts is well established, single cell transcriptome analyses are a relatively recent addition to the toolkit and the peak of its use and thereby its full potential might still be to come. Accordingly, the use of single cell transcriptomes is likely to continue to evolve with additional optimization of the chemistry involved and removal of current limitations, such as low read depth compared to bulk RNA sequencing. In this mini review, we are aiming to summarize the application of single cell transcriptome analyses and discuss the current use and overall potential of using zebrafish as a model for analyzing temporally dynamic developmental events. To illustrate the potential of zebrafish in SCT analysis we will focus on recent approaches taken to examine zebrafish ocular development.

### The approach of single cell transcriptome analyses

**FIGURE 1:**
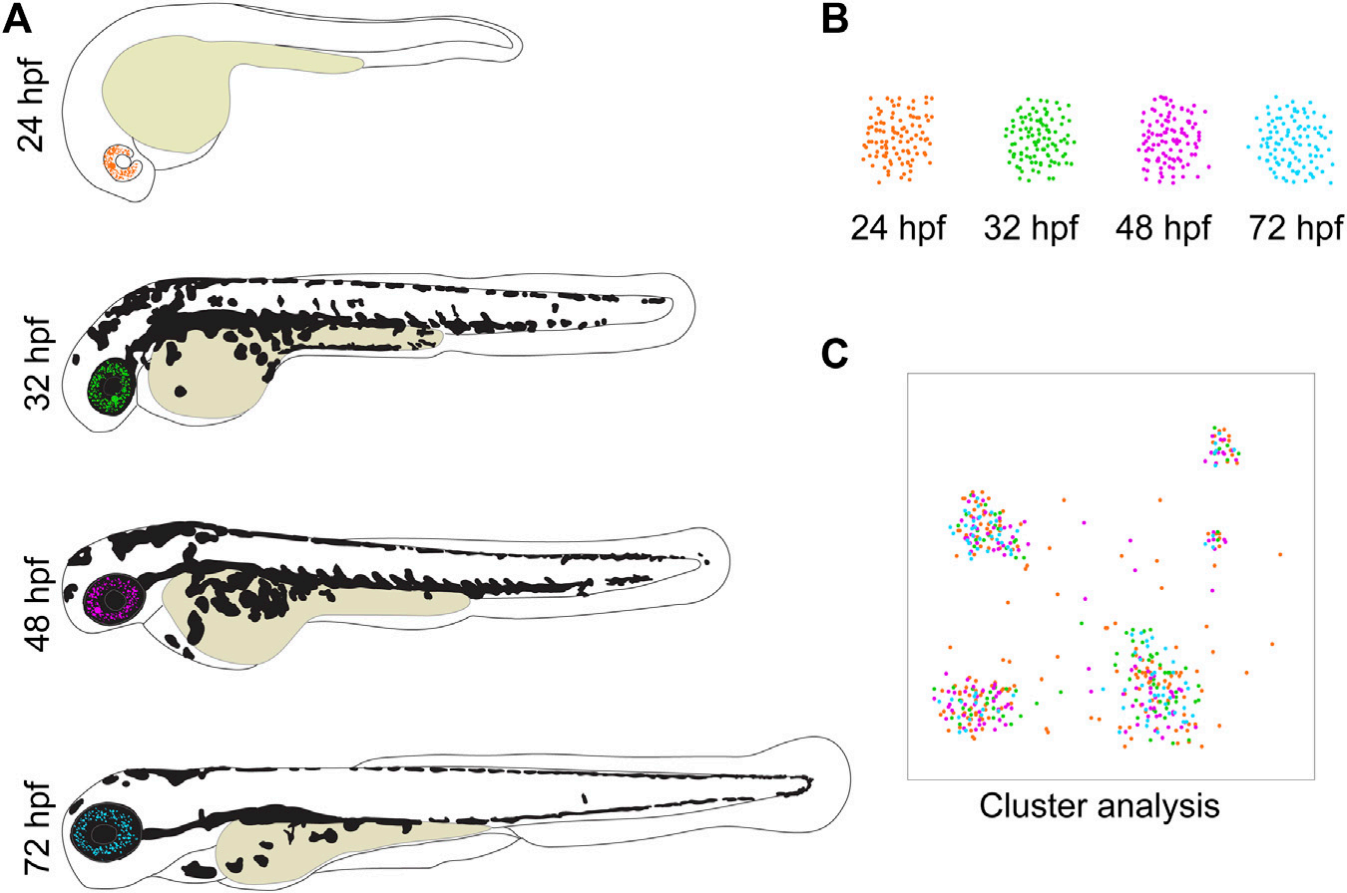
Zebrafish development as a model for eye tissues used for SCT analyses. **(A)** The transparency of zebrafish larvae makes them ideal to follow fluorescent cells throughout different stages of their development. **(B)** These cells can then easily be isolated by using FACS. Thereby, only the cells of interest are collected for analysis while non-desirable cells are sorted out. In this visualization, the cells from different developmental stages are depicted in different colors to highlight their origin. **(C)** The bioinformatical tools to analyze SCT data are progressing continuously and illustrated in a cartoon. A cluster analysis sorts the cells into groups according to their gene expression profiles, enabling the identification of present cell types. In this example, it is shown that clusters and thereby cell types can be composed of cells from different developmental time points.

Analyzing the transcriptome not only of whole tissues but of individual cells, opens completely new doors for understanding gene expression and its regulation. In the last few years, the application of this method exploded, ever increasing our knowledge about gene expression of specific tissues or cell types (Kanter and Kalisky, 2015; Xue et al., 2016; Farrell et al., 2018; Wagner et al., 2018; Adil et al., 2021; Mishra et al., 2022). In SCT analysis, individual cells are loaded onto a microfluidic chip and processed in such a fashion, that resulting cDNA libraries can retrospectively be assigned to a specific cell with high accuracy. Alternatively, a recently developed method named particle-templated instant partition sequencing (PIP-seq) avoids microfluid processing. Instead, it entails barcoding the cDNA after the cells were individually encapsulated in an emulsion (Clark et al., 2023). During the subsequent bioinformatical analysis, cells get sorted into groups, depending on their gene expression profiles (Figure 1). Thereby, we can examine individual and lineage associated cell expression from complex tissues. This leads to a detailed insight into the transcriptomic profiles of the studied tissues by applying current bioinformatics analysis tools like Seurat or Monocle that enable prediction of potential genetic interactions and regulatory networks (Trapnell et al., 2014; Satija et al., 2015). For example, in one of the pioneering studies, Wagner et al. pointed out the importance of chordin for the early development of the zebrafish larvae (Wagner et al., 2018). Ultimately, this can help unravel regulatory interactions and networks of genes crucial for cell development and maintenance. Naturally, this new information can prove to be highly useful when it comes to understanding and treating the occurrence of genetic disorders.

### The eye as a study object

Vision is one of the most fundamental processes in our daily life and vision impairment can have a negative impact on the quality of life. Further, the eye is of particular interest for SCT analysis for multiple reasons. It is a highly complex organ, composed of a variety of tissues that are in turn made of different cell types (Chow and Lang, 2001; Sinn and Wittbrodt, 2013; Miesfeld and Brown, 2019). The probably best-known part of the eye is the retina, the neuronal layer in the posterior segment of the eye. This layer alone already includes the photoreceptor cells (i.e., rods and cones), bipolar cells, horizontal cells, amacrine cells and retinal ganglion cells, which are necessary to detect light and initiate the signal transduction after light detection (Diacou et al., 2022). Non-retinal ocular components such as the often-neglected retinal pigment epithelium (RPE), or the anterior segment are also composed of multiple tissues including cornea, ciliary body, lens and trabecular meshwork which in turn require many specific cell types. All these cell types have their characteristic gene expression profiles and require specific interactions and regulations to differentiate. Moreover, the different components of the eye are derived from different embryonic tissues (Cvekl and Tamm, 2004; Soules and Link, 2005). For instance, while some components of the anterior segment are derived from periocular mesenchyme cells, others originate from cranial mesoderm. Thus, for a healthy eye to develop, these embryonic tissues must be orchestrated and organized by a fine-tuned network of interacting genes (Zuber et al., 2003; Sinn and Wittbrodt, 2013). A single mistake in this developmental process can lead to a severely negative impact on eye function and ultimately even lead to blindness. Furthermore, any attempts to engineer cells *in vitro* (via approaches such as iPSC) for the purpose of *in vivo* treatment will require precise understanding of the pathways involved in differentiation and specification. As such, detailed examination of early developmental stages is of particular interest to examine and characterize.

### Single cell transcriptome analysis in developmental biology

The popularity of SCT analyses has drastically increased within the last few years. This led to the identification of gene expression profiles for several different organ systems and cell types in different species (Klein et al., 2015; Briggs et al., 2018; Pandey et al., 2018; Cao et al., 2019). Novel types of neurons were specificized in the part of the zebrafish forebrain called habenular. The gene *nrp1a* was found to be specific for the left habenular and there was a general difference in left and right genetic profiles among the neurons (Pandey et al., 2018). Further, SCT analyses of *Xenopus tropicalis* showed that the onset of several cell types, such as endothelium, is earlier than previously assumed (Briggs et al., 2018). Also, direct comparison between bulk RNA sequencing and SCT analyses showed that the latter should be preferably used when studying individual cell types since the former cannot be as precise (Hegenbarth et al., 2022).

Analyses of retinal tissues alone have been so substantial that metadata analyses have already been performed singularly on retina specific data (Macosko et al., 2015; Welby et al., 2017; Phillips et al., 2018; Clark et al., 2019; Voigt et al., 2019; Lu et al., 2020; Swamy et al., 2021; Yi et al., 2021). The outcomes of these studies have demonstrated for instance, that cones cannot specify properly without the gene *atoh7* (Lu et al., 2020), gene expression of Müller glia cells differs among regions within the eye (Yi et al., 2021) and that NFI transcription factors play roles throughout eye development (Clark et al., 2019), pointing to the importance of SCT analyses. However, our understanding of eye development is far from complete. One of the issues with many popular model organisms is gathering sample tissue from multiple subsequent time points during development (Hegenbarth et al., 2022). The main limiting factors for this are the high costs of the SCT procedure and limited access to appropriate tissue, e.g., human donor organs (Welby et al., 2017; Phillips et al., 2018; Lukowski et al., 2019; van Zyl et al., 2022). Especially the limited access and ethical restrictions to embryonic human tissue are the main reasons, why transcriptome studies including early human development are still rare (Welby et al., 2017; Lu et al., 2020). While it can be expected that the general cost for SCT analyses will decrease over the next years, the problematic access to tissue of different developmental stages will remain a limiting factor. However, early developmental time points are of particular importance since gene expression at early stages can characterize the involvement of genes responsible for the early onset of diseases. In this respect zebrafish offer unique advantages.

### Advantages of using zebrafish for developmental single cell transcriptome analyses

In the following sections we aim to highlight the unique benefits that zebrafish offer when examining developmental eye transcriptomics including sample accessibility, precise tissue collection and functional analysis of candidate genes.

#### Sample accessibility

Many SCT studies analyzing ocular components, that do not include zebrafish, are limited to very few or only a single developmental stage, which is almost always fully mature (Shekhar et al., 2016; Phillips et al., 2018; Rheaume et al., 2018; Lukowski et al., 2019; Voigt et al., 2019; Yi et al., 2021; Ying et al., 2021; van Zyl et al., 2022). Further, it has already been pointed out that human donor tissue may already suffer from detrimental effects of old age (donor >60 years) or *postmortem* extraction, such as a reduced number of photoreceptor cells (Lukowski et al., 2019; Yi et al., 2021). Accordingly, the usefulness of these tissues to characterize gene expression regulation during early developmental processes may be limited. When compared to other model organisms, zebrafish are well-suited for the study of early developmental single cell transcriptomes (Parichy, 2015; Wagner et al., 2018; Teame et al., 2019; Farnsworth et al., 2020; Farnsworth et al., 2021; Gautam et al., 2021). The cultivation of zebrafish in the lab is more cost efficient than that of more popular models such as mice or rats. Most importantly, the morphological and physiological organization of the zebrafish eye and the regulatory signaling pathways are well conserved when compared to humans (>70% gene conservation), which makes them a great model to study human eye diseases (Morris, 2011; Gestri et al., 2012; Link and Collery, 2015; Santhanam et al., 2022). Zebrafish have external fertilization, a high fecundity rate (a single female can produce hundreds of embryos per week) and a rapid initial development, in which the essential components of the eye are developed by 5 days post fertilization (Cvekl and Tamm, 2004; Soules and Link, 2005). These advantages allow to easily access and collect high numbers of embryos of any desired developmental time point quickly without the necessity to surgically remove offspring from the mother. Thanks to external fertilization, it is also possible to monitor and regulate the pace of development (by adjusting the external temperature) which facilitates to access the exact stages of interest (Briggs et al., 2018; Farrell et al., 2018; Wagner et al., 2018; Farnsworth et al., 2020). A topic of particular interest is very early development, i.e., tracing cell development from the fertilized zygote to the early embryo. Two very detailed analyses have been conducted by Farrell and coworkers as well as Wagner and coworkers (Farrell et al., 2018; Wagner et al., 2018). Both studies minutely analyzed multiple developmental time points within the first 24 h and established the relationships of transcriptional trajectories of various cell types and states. Additionally, both compared gene expression patterns of mutant lines to those of wildtype fish. While Farrell and coworkers found that *nodal* mutants lacked mesendodermal cell types, Wagner and coworkers described an increase in ventral tissues in a *chordin* mutant line (Farrell et al., 2018; Wagner et al., 2018). Farnsworth and coworkers focused their attention to organogenesis of three early time points (1-, 2- and 5-days post fertilization), identifying over 200 cell clusters and highlighting gene expression changes of various cell types during early development (Farnsworth et al., 2020). Another approach has been to make use of the regenerative capabilities of zebrafish. For example, Celotto and coworkers induced injury to Müller glial cells in the retina and then traced the gene expression profile of these cells over several time points of their recovery. Thereby, they found that a subpopulation of Müller glial cells is involved in the renewal process of the recovering retina (Celotto et al., 2023). Clearly, zebrafish offer an unparallel access to large quantities of various tissues at any developmental timepoint.

#### Precise tissue collection

**FIGURE 2:**
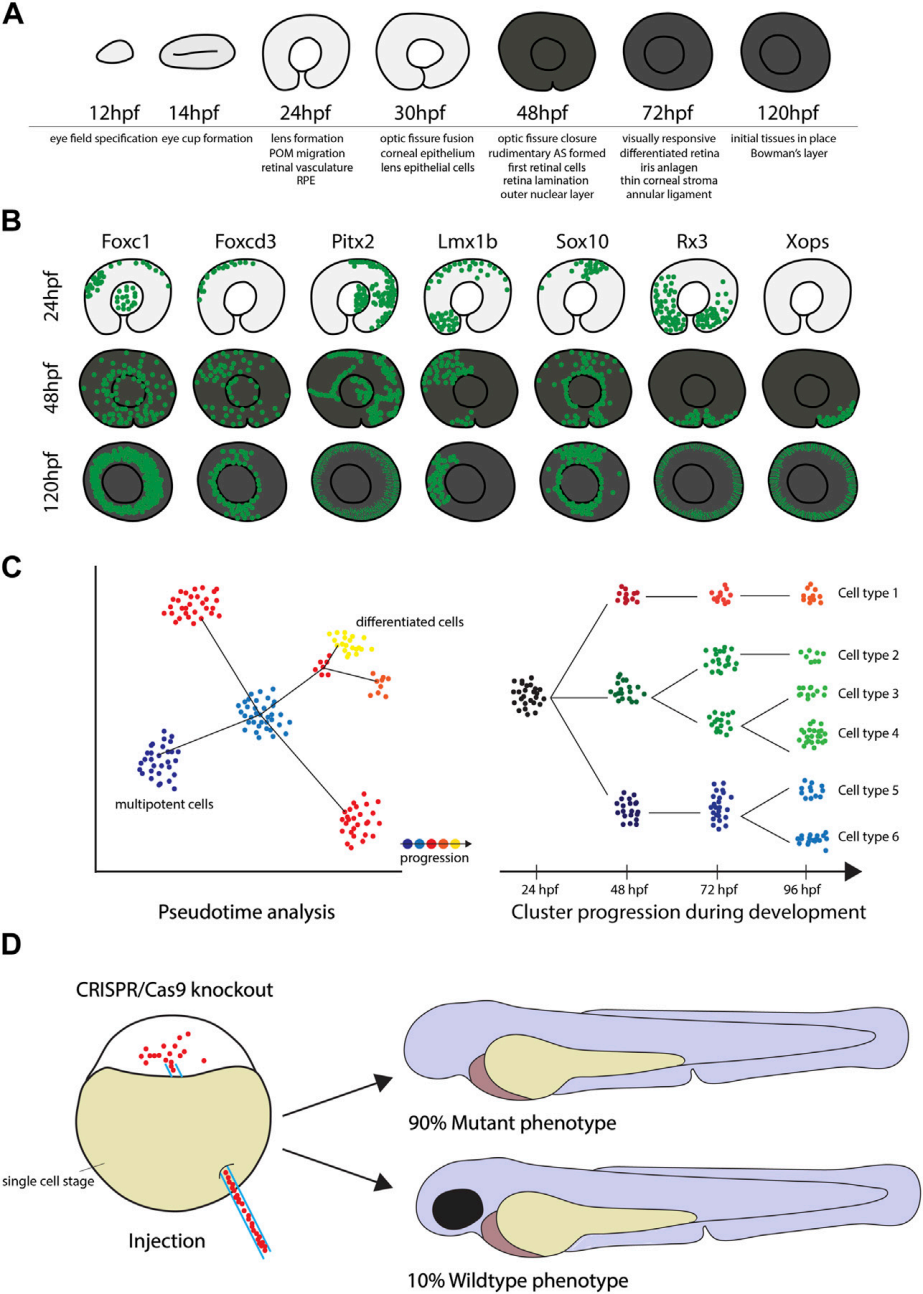
The use of zebrafish for Single Cell Transcriptome analyses. **(A)** The key stages of zebrafish eye development from 12 to 120hpf, including important milestones at the given timepoints. **(B)** Diagrams of GFP+ cells in the reporter lines Tg(*foxc1b*:GFP), Tg(*foxd3*:GFP), Tg(*pitx2*:GFP), Tg(*lmx1b*:GFP), Tg(*sox10*:GFP), Tg(*rx3*:GFP) and Tg(*xops*:GFP) display different migratory behavior and demonstrate that eye tissues have different developmental origins. **(C)** Time course SCT data can be analyzed in depth by using pseudotime analysis or cluster comparison as illustrated in the cartoon to determine changes in gene expression profiles throughout early stages of development. **(D)** CRISPR/Cas9 knockout efficiency in zebrafish can be as high as 90%.

Another critical aspect of SCT analyses is the excision of the tissue of interest. If the goal of the analysis is to study a specific tissue alone, it is imperative to include only this respective tissue in the analysis. The eye is a particularly delicate organ in which layers of different cell types are closely adjacent or intertwined. This can make manual isolation of individual cell types extremely difficult or even impossible. This increases the risk of accidently cross-contaminating samples and hence, misidentifying cells and potentially misinterpreting results. Also, due to the smaller eye size in small animal models, removing the correct tissue gets increasingly more challenging. Zebrafish offer a solution for these potential pitfalls, despite their own relatively small size. It is a well-established fact, that zebrafish are singularly suited when it comes to establishing transgenic lines. In these lines the expression of a fluorescent marker such as GFP or RFP is under the control of a promoter of a specific gene of interest. This way these transgenic lines can be used to solely represent a specific cell population of interest and study them in isolation. While these lines also exist for other model species such as mice, the expression of these fluorescent markers in transparent zebrafish larvae offers the unique possibility to trace fluorescent cells throughout development (Figures 1, 2). Well established transgenic lines useful for ocular developmental studies include those labeling the periocular mesenchyme, driven by promoters of: *foxc1b*, *foxd3*, *pitx2*, *lmx1b* and *sox10*, retinal marker *rx3* which progressively labels cells associated with the ventral region of the eye and the optic fissure as well as rod photoreceptor driver *XOPS* or cone photoreceptor driver *TαC* (Figure 2B) (Carmona et al., 2017; Hernandez et al., 2018; Van Der Meulen et al., 2020; Howard et al., 2021; Liu et al., 2022). For SCT analysis, this offers the unique possibility to narrow the analysis down to a specific cell type during a defined developmental time point as well as to avoid potential cross-contamination of the sample with cells from non-wanted tissues. With the help of fluorescent activated cell sorting (FACS), non-fluorescent cells can simply be sorted out, which allows the use of only the specifically desired cell type for the analysis. This method has already been applied successfully to isolate various neural crest cells including those forming the anterior segment, but also retinal cells, immune cells and cells of the developing gonads among others (see Figure 1) (Carmona et al., 2017; Hernandez et al., 2018; Zhang et al., 2019; Van Der Meulen et al., 2020; Howard et al., 2021; Tatarakis et al., 2021; Liu et al., 2022; Vöcking and Famulski, 2023). Hence, the dissection of the fragile tissue can be avoided altogether. The base requirement for this is to identify candidate genes whose expression is specific for a certain cell or tissue type (see Figure 2B). Establishing a reporter line with a fluorescent marker under the control of the promoter of this gene will then specifically mark only the desired cells. While not unique to zebrafish, this approach is standard in the zebrafish field and considered routine.

#### Exploring candidate gene function

Thanks to the ease of SCT analyses there is now a flood of potential eye development regulator candidate genes and the new challenge is to identify their function (Briggs et al., 2018; Farrell et al., 2018; Wagner et al., 2018; Clark et al., 2019; Farnsworth et al., 2020; Hoang et al., 2020; Farnsworth et al., 2021). For example, *klf7* was found to be important for the differentiation of retinal ganglion cells in different vertebrates (Gautam et al., 2021), while Voigt and co-workers pointed out the role of *bco2* for certain cone photoreceptors (Voigt et al., 2019). Due to the overwhelming abundance of candidates, characterizing actual gene functions is a looming problem that requires a solution. All too often in current SCT analyses, long lists of genes are given and a potential function in a certain tissue is claimed without further explanation or exploration of the actual gene function.

For functional analyses, zebrafish are again an ideal animal model. Before performing functional studies, the expression profile of the candidate gene can be easily and rapidly confirmed via whole mount *in situ* (WISH) hybridization experiments (Thisse and Thisse, 2008). Once a candidate gene with specific expression pattern has been identified, a great way to explore gene function is genetic knockout with the CRISPR system (Li et al., 2016; Choi et al., 2021). Particularly, the Alt R CRISPR/Cas9 system which has been demonstrated to be extremely effective in zebrafish, with a knockout success rate of up to 90% (Hoshijima et al., 2019). In fact, these injections can lead to bi-allelic cutting and therefore functional analysis in the F0 “crispants” (Figure 2D). This quick and direct knockout approach can also be supplemented with classical morpholino knockdowns (Walters et al., 2015). Either way, preliminary analysis, or screening, of potential candidates can be fast, inexpensive and even high throughput. These functional analyses are key not only to identify gene function, but also to characterize phenotypes resembling known ocular diseases (Choi et al., 2021; Santhanam et al., 2022). Accordingly, finding phenotypes created by genetic knockout might translate directly to known ocular diseases in humans.

### Zebrafish developmental single cell transcriptome application to study of anterior segment specification and assembly

Zebrafish have been well-established as a model for different human congenital ocular diseases such as coloboma, retinitis pigmentosa, glaucoma, holoprosencephaly and anterior segment dysgenesis (Morris, 2011; Gestri et al., 2012; Link and Collery, 2015; Hong and Luo, 2021; Santhanam et al., 2022). The general similarities in ocular development and genetic conservation make zebrafish a powerful model for the study of ocular disease (see Figure 2A). To date, most zebrafish-based studies have focused on retinopathies, but lately zebrafish are also being employed for the study of anterior segment dysgenesis (Gestri et al., 2012; Richardson et al., 2017; Hong and Luo, 2021; Seese et al., 2021; Santhanam et al., 2022; Vöcking and Famulski, 2023). Despite some differences in anatomy, our own group recently suggested that the annular ligament (AL) of zebrafish can be used as a model for the human trabecular meshwork, highlighting the potential use of zebrafish as a glaucoma model (Vöcking and Famulski, 2023). In this study, we collected anterior segment (AS) mesenchyme cells from several developmental time via FACS and analyzed expression in AS specific GFP + cells of the anterior segment reporter transgenic line Tg(*foxc1b*:GFP). Not only could we identify cell types of the cornea and AL, but we were also able to follow clusters of these cells throughout the different timepoints and thereby monitor changes in their gene expression profiles. We were able to compare our data to human studies (van Zyl et al., 2020; van Zyl et al., 2022) and found that human marker genes such as *krt4*, *krt5* and *myoc* are expressed in the zebrafish cornea and AL respectively. Moreover, we found previously unknown markers like *hgd* and *cndp1* expressed in the AL which might also play a role in human TM development (Vöcking and Famulski, 2023). This kind of multiple time point analyses enables the exact determination of the genetic profile of cells during their development and help identify critical moments of their differentiation (Figure 2C) (also see Farnsworth et al., 2020; Farnsworth et al., 2021). Since studies of human samples are mostly limited to mature tissues, insights of early timepoints in model organisms such as zebrafish are crucial to understand early development. Compared to other study animals, this approach is most feasible to perform in zebrafish due to ease of tissue collection and their affordability (Farrell et al., 2018; Wagner et al., 2018; Farnsworth et al., 2020; Farnsworth et al., 2021; Vöcking and Famulski, 2023).

## Conclusion

As we hope to summarize with this minireview, zebrafish are a nearly perfect model organism to use for multi-timepoint vertebrate developmental single cell transcriptome analysis and subsequent functional gene analysis.

Combining multiple developmental time points for single cell transcriptome analysis is a powerful tool to gain insights into developmental processes critical to the assembly of organs such as the eye. If this approach gets further combined with the use of transgenic lines, the examined cells can be limited to a specific group and their exact role during differentiation and specification. The use of zebrafish embryos facilitates all these advantages which makes the zebrafish embryo the ideal model. This has been nicely demonstrated by several recent studies, including our own, where different timepoints during development are used to highlight gene expression changes for cells involved in lens formation, anterior segment formation as well as differentiating retinal neurons (Farnsworth et al., 2020; Farnsworth et al., 2021; Vöcking and Famulski, 2023). Furthermore, using zebrafish offers the opportunity to easily study newly identified candidate genes via genetic knockout with the Alt R CRISPR/Cas9 system, as demonstrated by Farrell and coworkers (Farrell et al., 2018). Accordingly, we encourage the use of zebrafish at it provides unique opportunities and can therefore significantly increase the impact of developmental SCT studies in the eye or other major organ systems.
